# An incoherent feedforward loop formed by SirA/BarA, HilE and HilD is involved in controlling the growth cost of virulence factor expression by *Salmonella* Typhimurium

**DOI:** 10.1371/journal.ppat.1009630

**Published:** 2021-05-28

**Authors:** Deyanira Pérez-Morales, Jessica Nava-Galeana, Roberto Rosales-Reyes, Paige Teehan, Helen Yakhnin, Erika I. Melchy-Pérez, Yvonne Rosenstein, Miguel A. De la Cruz, Paul Babitzke, Víctor H. Bustamante

**Affiliations:** 1 Departamento de Microbiología Molecular, Instituto de Biotecnología, Universidad Nacional Autónoma de México, Cuernavaca, Morelos, México; 2 Consejo Nacional de Ciencia y Tecnología, Ciudad de México, México; 3 Unidad de Investigación en Medicina Experimental, Facultad de Medicina, Universidad Nacional Autónoma de México, Ciudad de México, México; 4 Department of Biochemistry and Molecular Biology, Center for RNA Molecular Biology, The Pennsylvania State University, University Park, Pennsylvania, United States of America; 5 Departamento de Medicina Molecular y Bioprocesos, Instituto de Biotecnología, Universidad Nacional Autónoma de México, Cuernavaca, Morelos, México; 6 Unidad de Investigación Médica en Enfermedades Infecciosas y Parasitarias, Hospital de Pediatría, Centro Médico Nacional Siglo XXI, Instituto Mexicano del Seguro Social, Ciudad de México, México; University of Toronto, CANADA

## Abstract

An intricate regulatory network controls the expression of *Salmonella* virulence genes. The transcriptional regulator HilD plays a central role in this network by controlling the expression of tens of genes mainly required for intestinal colonization. Accordingly, the expression/activity of HilD is highly regulated by multiple factors, such as the SirA/BarA two-component system and the Hcp-like protein HilE. SirA/BarA positively regulates translation of *hilD* mRNA through a regulatory cascade involving the small RNAs CsrB and CsrC, and the RNA-binding protein CsrA, whereas HilE inhibits HilD activity by protein-protein interaction. In this study, we show that SirA/BarA also positively regulates translation of *hilE* mRNA through the same mentioned regulatory cascade. Thus, our results reveal a paradoxical regulation exerted by SirA/BarA-Csr on HilD, which involves simultaneous opposite effects, direct positive control and indirect negative control through HilE. This kind of regulation is called an incoherent type-1 feedforward loop (I1-FFL), which is a motif present in certain regulatory networks and represents a complex biological problem to decipher. Interestingly, our results, together with those from a previous study, indicate that HilE, the repressor component of the I1-FFL reported here (I1-FFL_SirA/BarA-HilE-HilD_), is required to reduce the growth cost imposed by the expression of the genes regulated by HilD. Moreover, we and others found that HilE is necessary for successful intestinal colonization by *Salmonella*. Thus, these findings support that I1-FFL_SirA/BarA-HilE-HilD_ cooperates to control the precise amount and activity of HilD, for an appropriate balance between the growth cost and the virulence benefit generated by the expression of the genes induced by this regulator. I1-FFL_SirA/BarA-HilE-HilD_ represents a complex regulatory I1-FFL that involves multiple regulators acting at distinct levels of gene expression, as well as showing different connections to the rest of the regulatory network governing *Salmonella* virulence.

## Author summary

To infect the intestine of a broad range of hosts, including humans, *Salmonella* is required to express a large number of genes encoding different cellular functions, which imposes a growth penalty. Thus, *Salmonella* has developed complex regulatory mechanisms that control the expression of virulence genes. Here we identified a novel and sophisticated regulatory mechanism that is involved in the fine-tuned control of the expression level and activity of the transcriptional regulator HilD, for the appropriate balance between the growth cost and the virulence benefit generated by the expression of tens of *Salmonella* genes. This mechanism forms an incoherent type-1 feedforward loop (I1-FFL), which involves paradoxical regulation; that is, a regulatory factor exerting simultaneous opposite control (positive and negative) on another factor. I1-FFLs are present in regulatory networks of diverse organisms, from bacteria to humans, and represent a complex biological problem to decipher. Interestingly, the I1-FFL reported here is integrated by ancestral regulators and by regulators that *Salmonella* has acquired during evolution. Thus, our findings reveal a novel I1-FFL of bacteria, which is involved in virulence. Moreover, our results illustrate the integration of ancestral and acquired factors into a regulatory motif, which can lead to the expansion of regulatory networks.

## Introduction

Pathogenic bacteria have developed diverse regulatory mechanisms to control the appropriated spatiotemporal expression of virulence genes. Both activation and repression regulatory mechanisms are essential for bacteria to colonize different niches of hosts.

*Salmonella enterica* serovar Typhimurium (*S*. Typhimurium) contains a large number of virulence genes and a very complex regulatory network that control their expression. Two groups of genes mainly govern *Salmonella* virulence; those located in *Salmonella* Pathogenicity Island 1 (SPI-1) and those located in SPI-2. Both SPI-1 and SPI-2 encode a Type III Secretion System (T3SS), several effector proteins and chaperones, as well as transcriptional regulators [1,2]. The T3SS is a syringe-like multiprotein complex through which bacteria inject effector proteins directly into the cytoplasm of host cells. Effector proteins have distinct biological activities that alter different signal transduction pathways of eukaryotic host cells [3]. The SPI-1 genes mediate *Salmonella* invasion of cells at the intestinal epithelium, which generates enteritis, whereas the SPI-2 genes primarily mediate *Salmonella* replication within host cells [1,2]. Replication/survival inside macrophages within a membrane-bound compartment called the *Salmonella*-containing vacuole (SCV), leads to a systemic infection like typhoid fever [1,2]. Many other *Salmonella* virulence genes are located in distinct genomic regions, with their biological function being related to that of SPI-1 or SPI-2 [2,4,5].

Consistent with their role in intestinal infection, expression of the SPI-1 genes is induced when *Salmonella* resides in the intestinal lumen and in the cytosol of epithelial cells [6,7]. In addition, expression of the SPI-1 genes is regulated by different environmental cues commonly found in the intestine of hosts, such as short- and long-chain fatty acids, bile, oxygen level, osmolarity and pH [8–13]. *In vitro*, these genes are expressed in nutrient-rich media, such as lysogeny broth (LB), during the late exponential and early stationary growth phases [14–16]. Interestingly, different studies have reported that the SPI-1 genes show a bistable expression both *in vitro* [17–21] and in the gut lumen of mice [22], where only 10–50% of cells from clonal *Salmonella* populations express SPI-1. Moreover, it was shown that the two subpopulations of cells generated by this bistable expression (SPI-1^ON^ and SPI-1^OFF^) cooperate for the successful invasion of host cells and intestinal colonization by *Salmonella* [20,21].

Expression of the SPI-1 and related genes is controlled by a very complex regulatory network involving many positive and negative regulators that act at the transcriptional, translational or post-translational level [2,23,24]. The AraC-like regulator HilD, encoded in SPI-1, is the apex of different regulatory cascades through which HilD controls the expression of the SPI-1 genes and many other genes located outside of SPI-1. HilD induces expression of: 1) the SPI-1 genes and other genes located outside of SPI-1, through the HilA, InvF or SprB regulators, encoded in SPI-1 [2,23–26]; 2) the SPI-2 genes and other genes located outside SPI-2, through the SsrA/SsrB two-component system, encoded in SPI-2 [15,27,28]; 3) the flagellar and chemotaxis genes, through the FlhD_4_C_2_ transcriptional complex [29,30]; and 4) several other genes located in distinct genomic regions, by direct interaction [26,31,32]. Furthermore, HilD forms a positive feedforward regulatory loop with the AraC-like regulators HilC and RtsA, which recognize the same DNA motif as HilD and form heterodimers with HilD; however, HilD has a dominant function over HilC and RtsA [33–35].

Consistently with its role as a master regulator for *Salmonella* virulence, the expression, concentration and activity of HilD are tightly controlled, representing the central point for regulating SPI-1 genes and many other related genes [2,23,24]. For instance, the Hcp-like protein HilE, encoded in a genomic island other than the SPIs, inhibits HilD activity by protein-protein interaction, thereby affecting homodimerization and DNA binding of HilD [36–38]. In addition, the SirA/BarA two-component system induces expression of *hilD* at the translational level [39]. In this two-component system, SirA and BarA are the response regulator and the sensor kinase, respectively [40–42].

Orthologs of SirA/BarA are present in many other bacteria. In *Escherichia coli* (UvrY/BarA), *Pseudomonas* spp. (GacA/GacS), and *Vibrio cholerae* (VarA/VarS), this system controls expression of numerous genes encoding different cellular activities, including virulence, motility, biofilm formation and metabolism [43,44]. This system has been better characterized in *E*. *coli* where UvrY/BarA was shown to form a regulatory cascade with the non-translated small RNAs (sRNAs) CsrB and CsrC, and the RNA binding protein CsrA [44,45]. CsrA represses numerous genes at the translational level by interacting with sequences overlapping the ribosome binding sites on target mRNAs [44,45]. In response to its phosphorylation by BarA, UvrY directly activates transcription of CsrB and CsrC, which contain several binding sites for CsrA [44,45]. Thus, CsrB/CsrC sequester CsrA and antagonize its ability to regulate expression of target transcripts [44,45]. Several studies have shown that the counterparts of the UvrY/BarA-CsrB/CsrC-CsrA global regulatory network function similarly in other bacteria [43,44]. For instance, the SirA/BarA system induces expression of the SPI-1 genes through CsrB and CsrC, which antagonize the CsrA-mediated translational repression of the *hilD* transcript [39,46,47].

Interestingly, expression of SPI-1 genes causes growth retardation of *S*. Typhimurium in laboratory conditions. This effect becomes even more pronounced when the SPI-1 genes are overexpressed, such as in the absence of HilE [19]. However, it remains poorly understood how *S*. Typhimurium fine-tunes the expression level of the SPI-1 genes to maintain an appropriate balance between the penalty on growth and the benefit for virulence.

In this study, we report that the SirA/BarA-CsrB/CsrC regulatory cascade induces the expression of *hilE* by counteracting CsrA-mediated translational repression on the *hilE* mRNA, which reveals that SirA/BarA, HilD and HilE form an incoherent type-1 feedforward loop. Additionally, we demonstrate that HilE is necessary to reduce the growth cost imposed by the expression of genes regulated by HilD, in laboratory conditions and in the intestine of mice. Thus, our results support that the control of HilD expression by the feedforward loop formed by SirA/BarA, HilD and HilE, plays a role in the fitness of *S*. Typhimurium during intestinal infection of hosts.

## Results

### CsrA directly represses the expression of *hilE*

**Fig 1:**
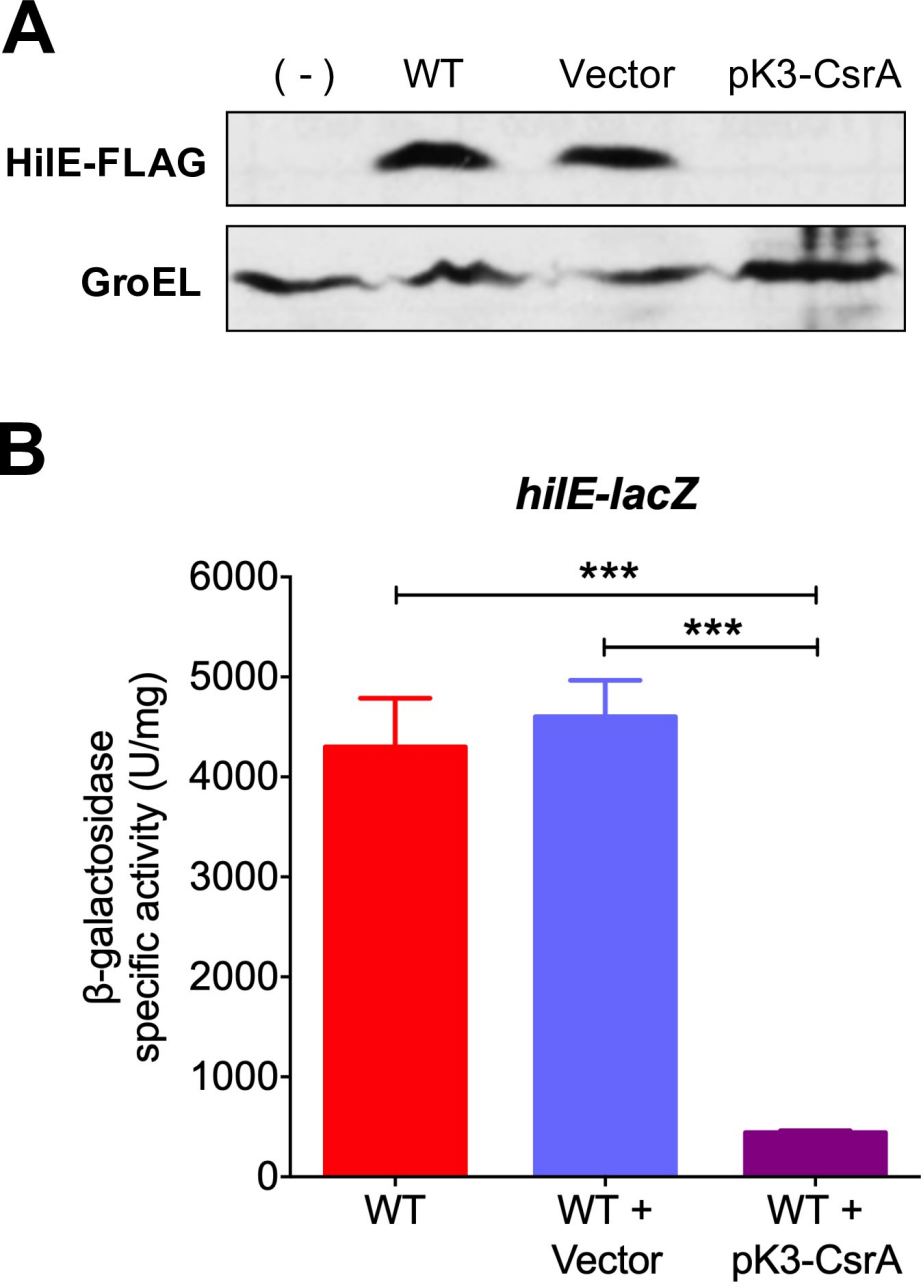
CsrA represses expression of *hilE*. (A) HilE-FLAG levels in the WT *S*. Typhimurium strain carrying a chromosomal FLAG-tagged *hilE* gene in the absence or presence of pMPM-K3 vector or the pK3-CsrA plasmid, which expresses CsrA from a constitutive promoter, were analyzed by Western blotting using monoclonal anti-FLAG antibodies. As a control for protein loading, the expression of GroEL was also determined using polyclonal anti-GroEL antibodies. A WT *S*. Typhimurium strain without the FLAG-tagged *hilE* gene was used as negative control (-). (B) β-galactosidase activity of the translational *hilE-lacZ* fusion contained in the philE-lacZ plasmid was determined in the WT *S*. Typhimurium strain in the absence or presence of pMPM-K3 vector or the pK3-CsrA plasmid. Data represent the average of three independent experiments done in duplicate. Error bars denote the standard deviations. Statistically different values are indicated (***, *p*-value < 0.0001). Western blot and β-galactosidase assays were performed with samples taken from bacterial cultures grown in LB at 37°C.

In a current project to analyze the global effect of CsrA on *Salmonella* we obtained circumstantial evidence that CsrA could negatively regulate the expression of *hilE*. To further investigate this phenomenon, we analyzed the chromosomal expression of FLAG-tagged HilE (HilE-FLAG) in the wild-type (WT) *S*. Typhimurium SL1344 strain carrying the pK3-CsrA plasmid expressing CsrA, when grown in conditions that favor the expression of SPI-1 genes (LB medium, at 37°C, with shaking; SPI-1-inducing conditions). Expression of CsrA from pK3-CsrA completely inhibited the amount of HilE-FLAG (Fig 1A). Similar results were obtained by assessing the expression of a *hilE-lacZ* translational fusion (Fig 1B). We were unable to evaluate the expression of HilE-FLAG and *hilE-lacZ* in the absence of CsrA because *csrA* mutants of *S*. Typhimurium exhibit severe growth defects [39,48]. These results indicate that CsrA negatively controls the expression of *hilE*.

**Fig 2:**
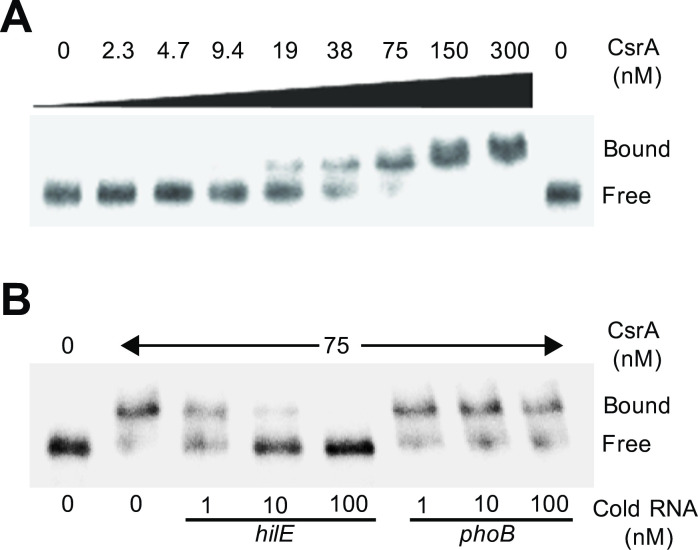
CsrA specifically interacts with the leader region of the *hilE* transcript. (A) CsrA binding to the *hilE* RNA was analyzed using EMSAs by incubating labeled *hilE* RNA (0.2 nM) with increasing concentrations of purified CsrA (0, 2.3, 4.7, 9.4, 19, 38, 75, 150 and 300 nM). Positions of bound and free RNA are marked. These experiments were performed three times and a representative gel is shown. (B) RNA competition experiment where labeled *hilE* RNA (0.2 nM) was combined with 1, 10 and 100 nM of unlabeled specific (*hilE*) or non-specific (*phoB*) competitor RNA and incubated with 0 and 75 nM of purified CsrA. Positions of bound and free RNA are marked. A representative gel from two independent assays is shown.

To determine whether CsrA regulates *hilE* directly, quantitative electrophoretic mobility shift assays (EMSAs) were performed using purified CsrA and the 5’-end-labelled leader RNA of *hilE*. A band with lower mobility was detected with concentrations of CsrA between 19 and 75 nM, indicating that CsrA formed a complex with the *hilE* transcript; at 150 and 300 nM CsrA a second complex with even lower mobility was also observed (Fig 2A). These data support that CsrA binds two sites on the *hilE* transcript. Nonlinear least-squares analysis of these EMSAs data yielded an apparent *K*_*d*_ value of 37 ± 13 nM CsrA for *hilE* mRNA. The specificity of the CsrA-*hilE* RNA interaction was evaluated by performing competition experiments with specific (*hilE*) and non-specific (*phoB*) unlabeled RNA competitors. Whereas unlabeled *hilE* RNA was an effective competitor, *phoB* RNA was not (Fig 2B). In agreement with these results, data from a previous global analysis by CLIP-seq showed that CsrA binds *in vivo* to a sequence located near the translation start codon of the *hilE* mRNA [49]. Thus, we conclude that CsrA binds specifically to the *hilE* leader transcript. Together, our results indicate that CsrA directly represses the expression of *hilE*.

### SirA/BarA induces the expression of *hilE* through CsrB/C

**Fig 3:**
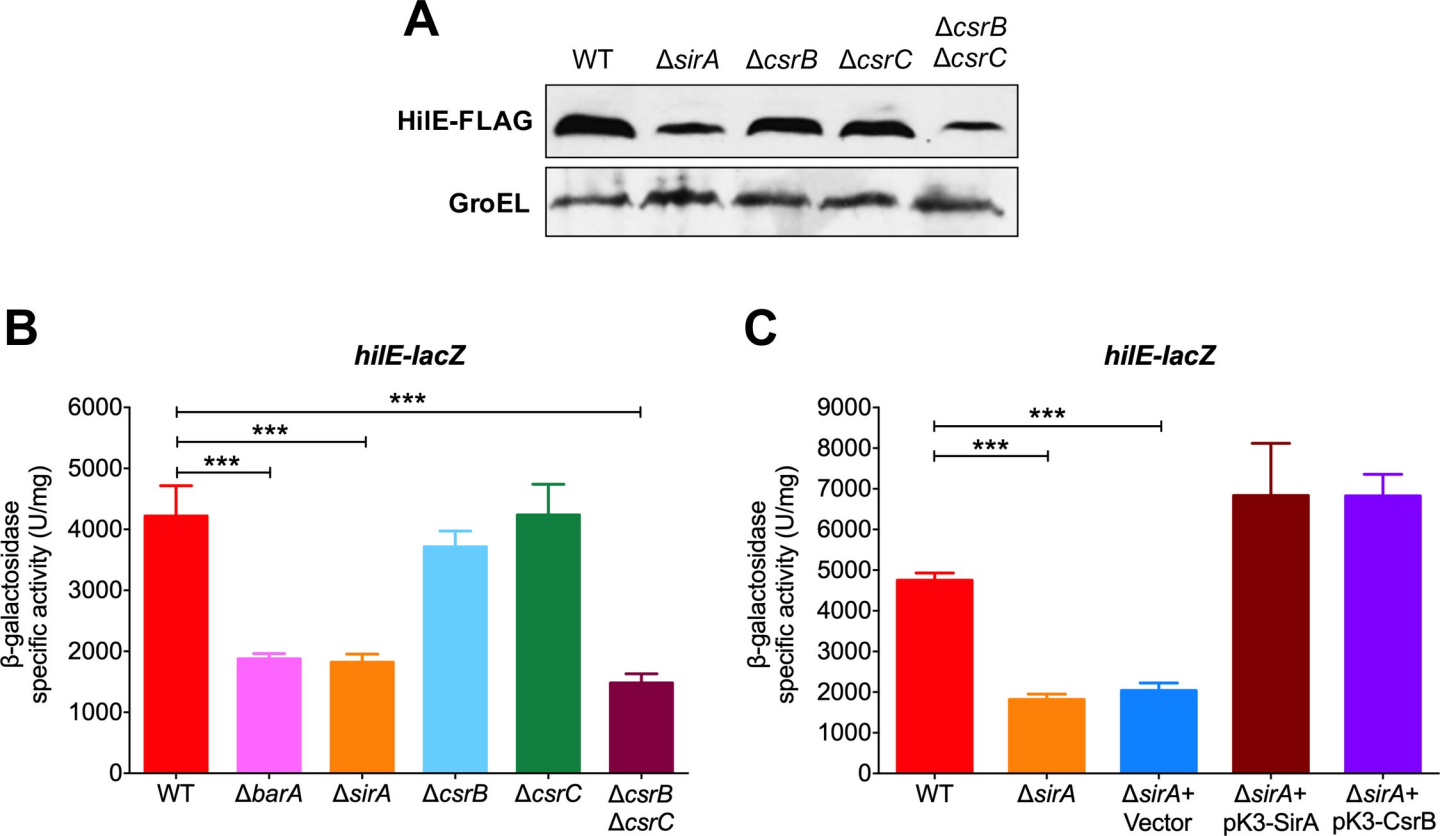
SirA/BarA and CsrB/CsrC positively regulate the expression of *hilE*. (A) Western blot analysis of HilE-FLAG expression in the WT *S*. Typhimurium strain and its derivative Δ*sirA*, Δ*csrB*, Δ*csrC* and Δ*csrB* Δ*csrC* mutants carrying a chromosomal FLAG-tagged *hilE* gene, using monoclonal anti-FLAG antibodies. As a protein loading control, the expression of GroEL was also determined using polyclonal anti-GroEL antibodies. (B) β-galactosidase activity of the translational *hilE-lacZ* fusion contained in the philE-lacZ plasmid was determined in the WT *S*. Typhimurium strain and its derivative Δ*barA*, Δ*sirA*, Δ*csrB*, Δ*csrC* and Δ*csrB* Δ*csrC* mutants. (C) β-galactosidase activity of the translational *hilE-lacZ* fusion contained in the philE-lacZ plasmid was determined in the WT *S*. Typhimurium strain and its derivative Δ*sirA* mutant in the absence or presence of pMPM-K3 vector, pK3-SirA or pK3-CsrB plasmids, which express SirA and CsrB, respectively, from a constitutive promoter. Data represent the average of three independent experiments done in duplicate. Error bars symbolize the standard deviations. Statistically different values are indicated (***, *p*-value < 0.0001). Western blot and β-galactosidase assays were performed with samples taken from bacterial cultures grown in LB at 37°C.

CsrA-mediated repression of target genes is counteracted by the two-component system SirA (UvrY)/BarA through the sRNAs CsrB and CsrC, which bind and sequester CsrA [44,45]. To define the complete regulatory cascade involving CsrA that controls the expression of *hilE*, the expression of HilE-FLAG and that of the *lacZ-hilE* fusion was monitored in the WT *S*. Typhimurium strain and its Δ*sirA*, Δ*csrB*, Δ*csrC* and Δ*csrB* Δ*csrC* derivative mutants, grown in SPI-1-inducing conditions. Both the amount of HilE-FLAG and the expression of *hilE-lacZ* were reduced in the Δ*sirA* mutant, as well as in the Δ*csrB* Δ*csrC* double mutant, compared with the WT strain (Fig 3A and 3B). Additionally, as shown in Fig 3B, activity of the *hilE-lacZ* fusion was reduced in the Δ*barA* mutant lacking BarA, the cognate sensor kinase of the SirA response regulator [41,42]. In contrast, the Δ*csrB* and Δ*csrC* single mutants showed WT levels of HilE-FLAG and *hilE-lacZ* expression (Fig 3A and 3B), which is consistent with previous reports indicating that in *S*. Typhimurium, the absence of both CsrB and CsrC is required for observable effects on the expression of other target genes of the SirA/BarA-Csr cascade [39,50]. As expected, complementation of the Δ*sirA* mutant with the pK3-SirA plasmid expressing SirA, restored the activity of the *hilE-lacZ* fusion to levels even slightly higher than those of the WT strain (Fig 3C). Moreover, complementation of the Δ*sirA* mutant with the pK3-CsrB plasmid expressing CsrB, also restored the activity of the *hilE-lacZ* fusion (Fig 3C). Collectively, these results indicate that the SirA/BarA two-component system induces the expression of *hilE* through the sRNAs CsrB and CsrC, which counteract repression of this gene by CsrA.

### HilE represses HilD-mediated expression of SPI-1, SPI-2 and SPI-5 genes

**Fig 4:**
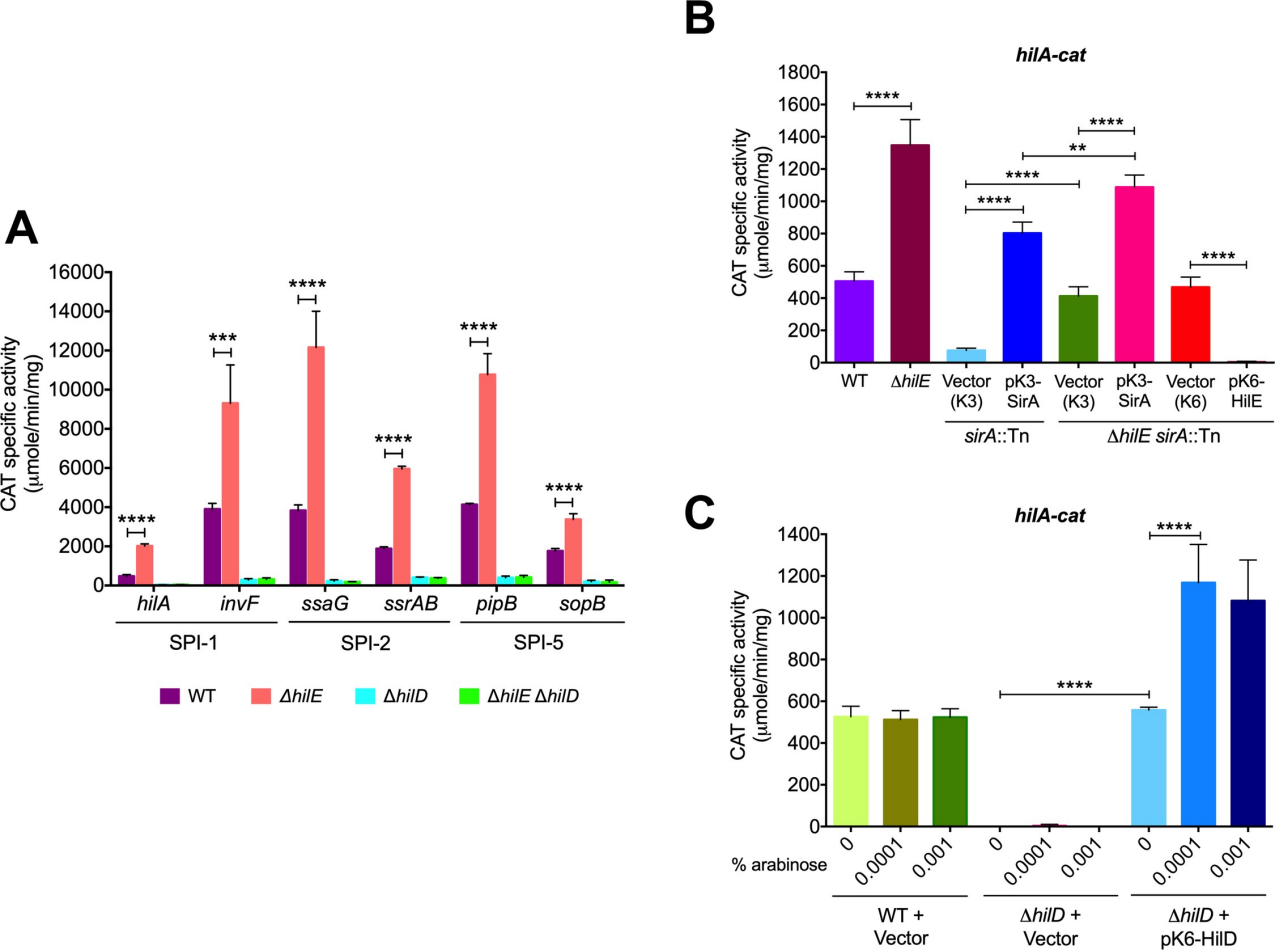
HilE represses the expression of genes regulated by HilD. (A) CAT activity of the *hilA-cat*, *invF-cat*, *ssaG-cat*, *ssrAB-cat*, *pipB-cat* and *sopB-cat* transcriptional fusions, contained in plasmids philA-cat, pinvF-cat, pssaG-cat, pssrAB-cat, ppipB-cat and psopB-cat, respectively, was determined in the WT *S*. Typhimurium strain and its derivative Δ*hilE*, Δ*hilD* and Δ*hilE* Δ*hilD* mutants. (B) CAT activity of the *hilA-cat* transcriptional fusion, contained in the philA-cat plasmid, was determined in the WT *S*. Typhimurium strain and its derivative Δ*hilE*, *sirA*::Tn*10*d and Δ*hilE sirA*::Tn*10*d mutants in the absence or presence of pMPM-K3 or pMPM-K6 vectors, the pK3-SirA plasmid expressing SirA from a constitutive promoter or the pK6-HilE plasmid expressing HilE from an arabinose-inducible promoter. Expression of HilE from pK6-HilE was induced by adding 0.001% L-arabinose to the medium. (C) CAT activity of the *hilA-cat* transcriptional fusion, contained in the philA-cat plasmid, was determined in the WT *S*. Typhimurium strain and its derivative Δ*hilD* mutant carrying the pMPM-K6 vector or the pK6-HilD plasmid, which expresses HilD from an arabinose-inducible promoter. Expression of HilD from pK6-HilD was induced with 0%, 0.0001% or 0.001% L-arabinose. CAT-specific activity was obtained from samples collected from bacterial cultures grown in LB at 37°C. Data represent the average of three independent experiments done in duplicate. Error bars indicate the standard deviations. Statistically different values are indicated (**, *p*-value < 0.01; ***, *p*-value < 0.001; ****, *p*-value < 0.0001).

HilE negatively affects the activity of the transcriptional regulator HilD [36–38]. Consequently, HilE would decrease the expression of the large number of genes controlled by HilD. To further investigate this repressor role of HilE, we quantified the expression of two genes each from SPI-1 (*hilA* and *invF*), SPI-2 (*ssrAB* and *ssaG*) and SPI-5 (*sopB* and *pipB*), as *cat* transcriptional fusions. HilD positively regulates the expression of all of these genes directly or indirectly [15,23,27,33,51–53]. Experiments were performed in the WT *S*. Typhimurium strain and its Δ*hilE* derivative mutant grown in SPI-1-inducing conditions. As shown in Fig 4A, the expression of every gene increased significantly in the Δ*hilE* mutant compared with the WT strain. As expected, due to the absence of HilD, only low expression level was observed for these genes in the Δ*hilD* and Δ*hilE* Δ*hilD* mutants (Fig 4A). Consistent with our results, previous studies also indicate that the expression of *hilA* increases in the absence of HilE [36,54]. Additionally, we examined and compared the expression level of the *hilA-cat* fusion in the WT *S*. Typhimurium strain and its Δ*hilE*, *sirA*::Tn*10*d (transposon insertion in *sirA*) and Δ*hilE sirA*::Tn*10*d derivative mutants in the absence or presence of the pK3-SirA plasmid expressing SirA, the pMPM-K3/K6 vector, or the pK6-HilE plasmid expressing HilE. In agreement with previous studies indicating that SirA positively regulates SPI-1 genes [39,40,42,55], the *hilA-cat* fusion showed reduced expression levels in the *sirA*::Tn*10*d mutant containing the pMPM-K3 vector, with respect to the WT strain (Fig 4B). Surprisingly, the *hilA-cat* fusion was expressed in the Δ*hilE sirA*::Tn*10*d double mutant containing the pMPM-K3 vector at a level only slightly lower than in the WT strain (Fig 4B), revealing than in the absence of HilE, *hilA* is expressed independently of SirA. It is important to consider that the HilE expression is positively controlled by SirA (this study) but also by other regulators [56–59] as discussed later. In fact, our results show that a partial amount of HilE is present in the absence of SirA (Fig 3). Thus, our results suggest that in the absence of SirA the expression of *hilA* is repressed by two mechanisms: 1) CsrA inhibits translation of the *hilD* mRNA and 2) the diminished amount of HilE is able to inactivate background levels of HilD, which would avoid the positive autoregulation of HilD. It is reasonable to suggest that when SirA counteracts the CsrA-mediated repression of *hilD*, higher levels of HilE would be required to negatively control the activity of HilD, which can be reached with the positive regulation of the *hilE* expression by SirA.

The presence of the pK3-SirA plasmid further increased the expression of *hilA-cat* in the Δ*hilE sirA*::Tn*10*d mutant and, as could be expected, the presence of the pK6-HilE plasmid completely inhibited the expression of *hilA-cat* in the Δ*hilE sirA*::Tn*10*d mutant (Fig 4B). To note, the *hilA-cat* fusion showed similar expression levels in the Δ*hilE* and Δ*hilE sirA*::Tn*10*d + pK3-SirA mutants, as well as in the Δ*hilD* mutant complemented with the pK6-HilD plasmid overexpressing HilD (Fig 4B and 4C), which could suggest that in the absence of HilE (or overexpression of HilD) maximal expression levels of *hilA* are reached.

**Fig 5:**
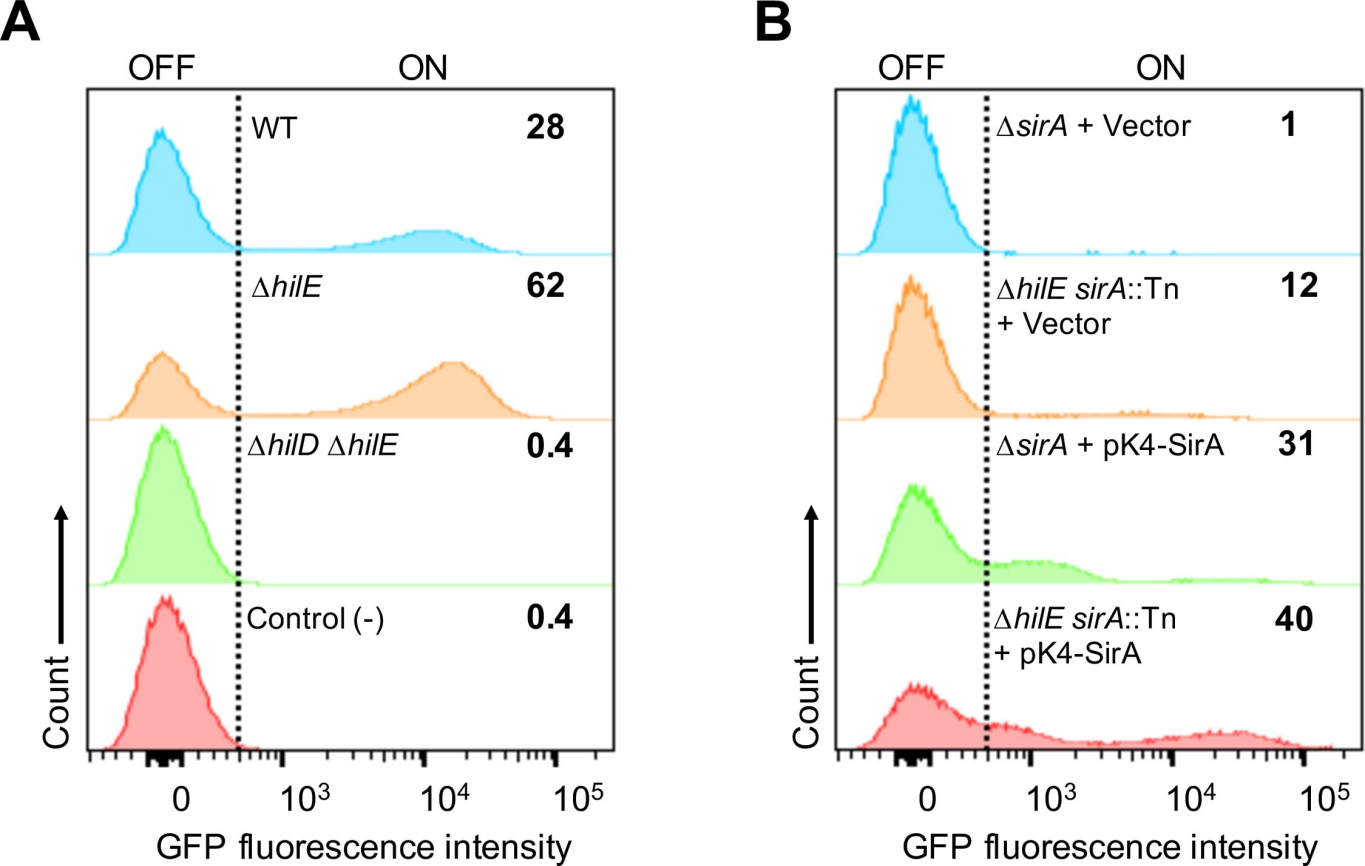
HilE negatively controls the bistable expression of SPI-1. Flow cytometry analysis of GFP fluorescence expression of the *invF-gfp* transcriptional fusion contained in the low copy pMPMA3ΔPlac P*invF*-*gfp*[LVA]/R plasmid in different *S*. Typhimurium strains. (A) GFP expression in cultures of the WT strain and its derivative Δ*hilE* and Δ*hilE* Δ*hilD* mutants carrying the *invF-gfp* fusion, as well as in cultures of the WT strain carrying the *gfp* reporter gene without a promoter (negative control). (B) GFP expression in cultures of the *sirA*::Tn*10*d and Δ*hilE sirA*::Tn*10*d mutants carrying the *invF-gfp* fusion and the pMPM-K4Ω vector or the pK4-SirA plasmid expressing SirA. The percentage of the GFP+ population of each strain is indicated on the graphs. Data shown are representative of two independent experiments performed with ten independent bacterial colonies.

We next aimed to analyze the effect of HilE on the fraction of cells expressing SPI-1 genes in the *Salmonella* population. To do this, GFP fluorescence expression from an *invF-gfp* transcriptional fusion was quantified by flow cytometry in cultures of the WT *S*. Typhimurium strain and its Δ*hilE* and Δ*hilE* Δ*hilD* derivative mutants, as well as in cultures of the *sirA*::Tn*10*d and Δ*hilE sirA*::Tn*10*d mutants carrying the pK4-SirA plasmid expressing SirA or the pMPM-K4Ω vector, grown in SPI-1-inducing conditions. Similar to other reports [17–21], only 28% of the cells from cultures of the WT strain expressed *invF-gfp*, whereas 62% of the cells expressed this fusion in cultures of the Δ*hilE* mutant (Fig 5A). As expected, expression of *invF-gfp* in the Δ*hilE* Δ*hilD* mutant or the WT strain carrying the *gfp* reporter gene without a promoter showed nearly undetectable expression of GFP fluorescence (Fig 5A). In addition, the fraction of cells expressing the *invF-gfp* fusion was higher in the Δ*hilE sirA*::Tn*10*d double mutant than in the *sirA*::Tn*10*d single mutant, both in the presence of the vector pMPM-K4Ω or the pK4-SirA plasmid (Fig 5B). Intriguingly, complementation of the *sirA*::Tn*10*d and Δ*hilE sirA*::Tn*10*d mutants yielded a subpopulation of cells with an *invF-gfp* expression pattern somewhat different to that of cells from the WT strain and the Δ*hilE* mutant (Fig 5A and 5B), which seems to be an effect of the expression of SirA from a multicopy plasmid. Nevertheless, these results clearly show that HilE reduces the fraction of cells expressing SPI-1 genes in the *Salmonella* population.

Collectively, these results reinforce that HilE plays a major role in regulating the SPI-1 genes by acting as a repressor of HilD. It is important to note that the presence of HilE only decreases, but does not eliminate, the HilD-mediated activation of target genes, or the fraction of cells from the *Salmonella* population that expresses these genes, in SPI-1-inducing conditions.

### HilE reduces the deleterious effect on growth caused by expression of genes controlled by HilD

Interestingly, SirA/BarA induces the expression of both HilD [39] and HilE (this study), which would seem counterintuitive. What would be the role of the regulation of *hilE* by SirA/BarA?

**Fig 6:**
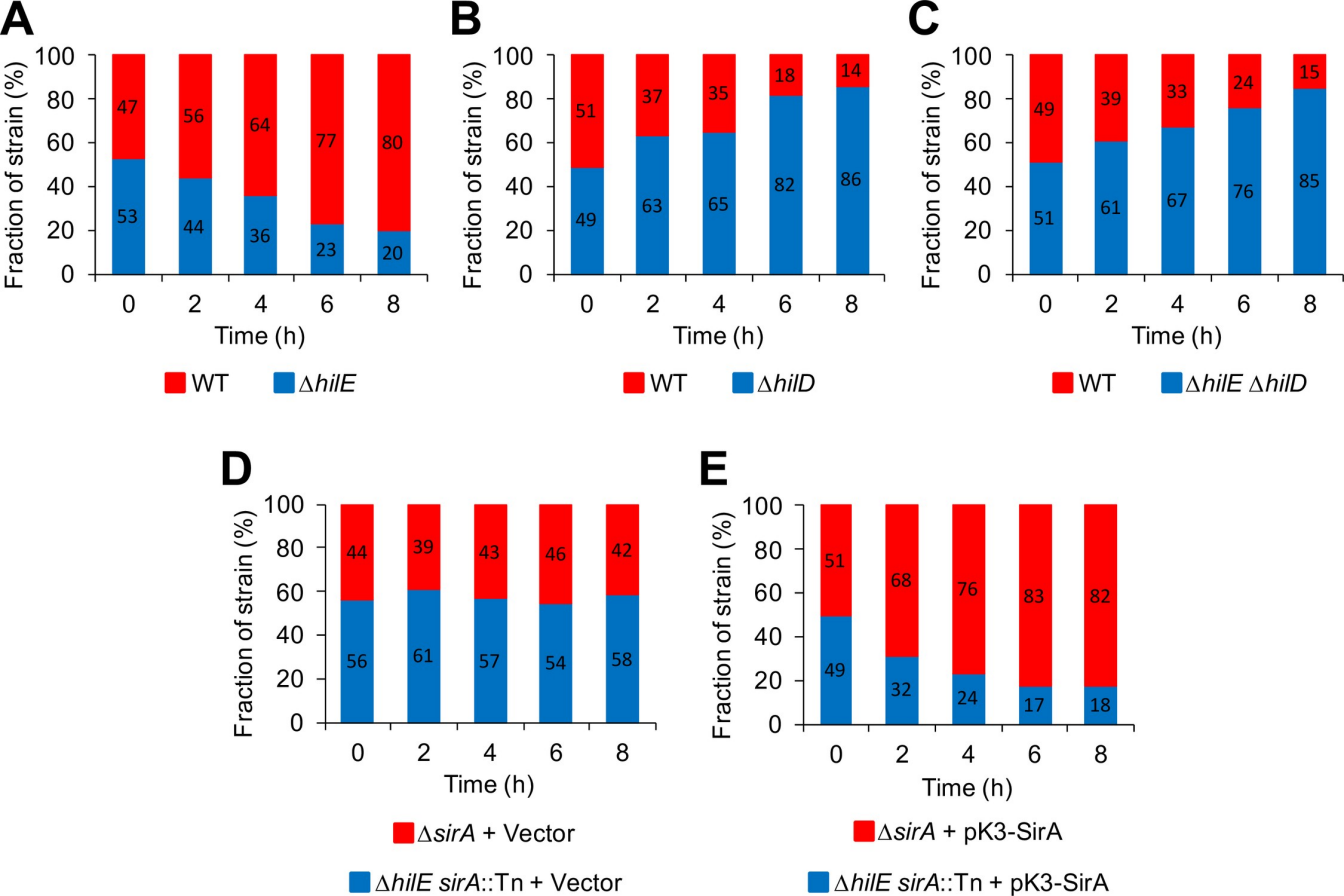
Detrimental effect on bacterial growth by the expression of genes controlled by HilD. *In vitro* competitive growth (A) between WT *S*. Typhimurium strain and isogenic Δ*hilE* mutant, (B) between WT *S*. Typhimurium strain and isogenic Δ*hilD* mutant, (C) between WT *S*. Typhimurium strain and isogenic Δ*hilE* Δ*hilD* mutant, (D) between the Δ*sirA* and Δ*hilE sirA*::Tn*10*d mutants carrying the pMPM-K3 vector, and (E) between the Δ*sirA* and Δ*hilE sirA*::Tn*10*d mutants carrying the pK3-SirA plasmid expressing SirA, was determined by counting CFUs for each strain from samples taken at 0, 2, 4, 6 and 8 h of bacterial mixed cultures grown in LB at 37°C with shaking. CFUs are represented as fraction (percentage) of each strain in the respective mixed culture. Data represent the average of three independent experiments.

Overexpression of HilD or the absence of HilE retard growth of *S*. Typhimurium in competitive assays performed in LB, which was partially attributable to the overexpression of some genes regulated by HilD [19]. To further investigate this phenomenon, we monitored the growth of the WT *S*. Typhimurium strain and the Δ*hilE*, Δ*hilD* and Δ*hilE* Δ*hilD* mutants, in mixed cultures grown in SPI-1-inducing conditions. In the WT/Δ*hilE* mixed cultures, the WT strain outcompeted the Δ*hilE* mutant, representing 80% of the viable cells at the end of these assays (Fig 6A). In contrast, in the WT/Δ*hilD* mixed cultures, the WT strain was outcompeted by the Δ*hilD* mutant; only 14% of the viable cells at the end of these assays were WT (Fig 6B). These results are in agreement with those obtained previously, showing that the absence of HilE and HilD has negative and positive effects, respectively, on the growth of *S*. Typhimurium cultures [19]. Furthermore, we observed that the WT strain was also outcompeted by the Δ*hilE* Δ*hilD* mutant to a similar extent as the Δ*hilD* single mutant (Fig 6B and 6C), suggesting that the negative effect caused by the absence of HilE on the *S*. Typhimurium growth requires the presence of HilD.

Additionally, we analyzed the competitive growth between the Δ*sirA* and Δ*hilE sirA*::Tn*10*d mutants carrying the pMPM-K3 vector or the pK3-SirA plasmid expressing SirA. Interestingly, in the presence of the pMPM-K3 vector, the Δ*sirA* and Δ*hilE sirA*::Tn*10*d mutants grew similarly through the time assessed (Fig 6D). In contrast, in the presence of the pK3-SirA plasmid, the Δ*hilE sirA*::Tn*10*d mutant was outcompeted by the Δ*sirA* mutant; only 18% of the viable cells at the end of these assays were from the Δ*hilE sirA*::Tn*10*d mutant strain (Fig 6E). These results reveal that SirA is required for HilE to play a role in the fitness of *S*. Typhimurium.

Together, these results show that HilE reduces the deleterious effect on the *S*. Typhimurium growth caused by the expression of genes regulated by HilD in laboratory conditions.

**Fig 7:**
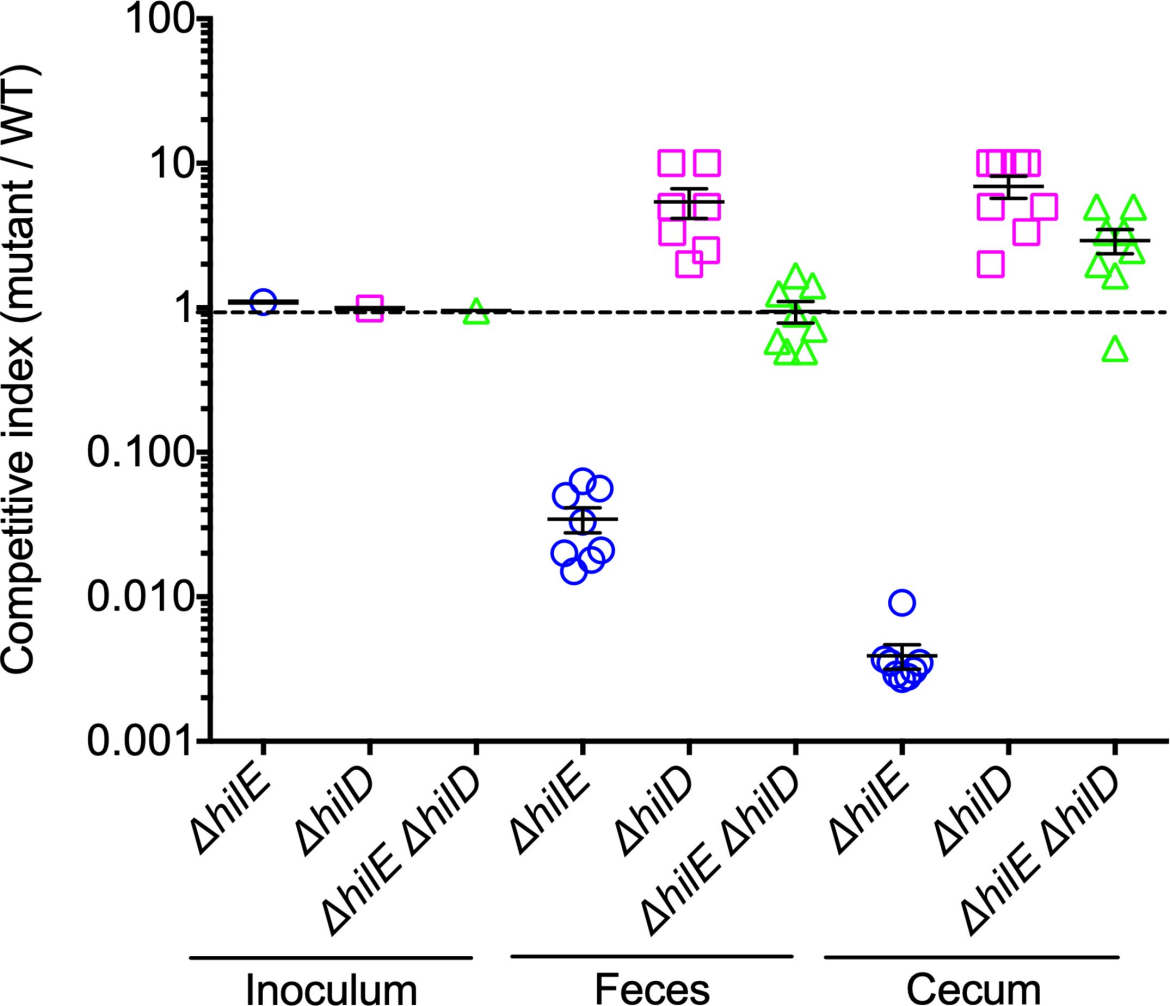
HilE is required for the intestinal colonization of mice by *S*. Typhimurium. Groups of eight streptomycin-pretreated mice were orally inoculated with a mixed bacterial suspension containing an equal amount of CFUs (0.5 x 10^6^) of the WT *S*. Typhimurium strain and the respective mutant (WT + Δ*hilE*, WT + Δ*hilD* or WT + Δ*hilE* Δ*hilD*). At 3 days post-infection, CFUs from feces and cecum were counted for each strain and a Competitive Index (CI; CFUs mutant / CFUs WT) was obtained. A CI was also determined for the respective inoculum. Data for each mouse or inoculum were log-transformed and plotted. CIs are indicated as follows: WT/Δ*hilE*, blue circles; WT/Δ*hilD*, pink squares; and WT/Δ*hilE* Δ*hilD*, green triangles. Bars denote the standard error of the mean for each experimental group. CI of 1.0, represented by a horizontal dotted line, indicates that the two strains (WT and mutant) were present in equivalent numbers.

SPI-1-inducing conditions are considered to somehow mimic the intestinal environment found by *Salmonella* in hosts. Therefore, on the basis of our results described above, we investigated whether HilE is important for successful intestinal colonization by *S*. Typhimurium. For this analysis, we determined and compared the survival of the WT *S*. Typhimurium strain to that of the Δ*hilE*, Δ*hilD* and Δ*hilE* Δ*hilD* mutants, in the intestine of streptomycin-pretreated mice, a model used to study the intestinal colonization by *S*. Typhimurium [60]. Groups of eight mice were orally infected with a mix of an equal number of cells of the WT strain and each mutant strain. Three days post-infection, bacteria from feces and cecum of mice were counted, and a competitive index was obtained to determine the proportion between each mutant and the WT strain. The Δ*hilE* mutant showed a ~30-fold and ~250-fold reduction in survival with respect to the WT strain, in feces and cecum, respectively. In contrast, the survival of the Δ*hilD* and Δ*hilE* Δ*hilD* mutants was similar or slightly higher than that of the WT strain in both feces and cecum (Fig 7). Thus, the absence of HilE attenuates the intestinal colonization by *S*. Typhimurium in a HilD-dependent way. These results are in agreement with those from a previous study showing that HilE is important for the intestinal disease caused by long term infections of *S*. Typhimurium in mice (i.e. at 10 days post-infection), including colonization and induction of enteritis [20]. Furthermore, our results are also consistent with reports indicating that SPI-1 or HilD function is required to invade cells from the intestinal epithelium, and thus to induce enteritis, but not to colonize the cecum lumen [2,20,60–62]. Replication of *Salmonella* in the intestinal lumen has been shown to require genes for the use of specific nutrients present in the inflamed intestine, such as the *eut*, *ttr* and *pdu* genes [63–65], which, according to previous transcriptomic analyses, are not regulated by HilD [66]. These data reveal a crucial role for HilE during intestinal infection by *S*. Typhimurium.

In all, together with previous reports, our data suggest that by inducing the expression of HilD and its negative regulator HilE, SirA/BarA exerts a fine-tuning regulatory control that cooperates to lessen the growth penalty produced by HilD-mediated expression of several tens of virulence factors, which is required for the successful intestinal colonization by *S*. Typhimiurium.

## Discussion

**Fig 8:**
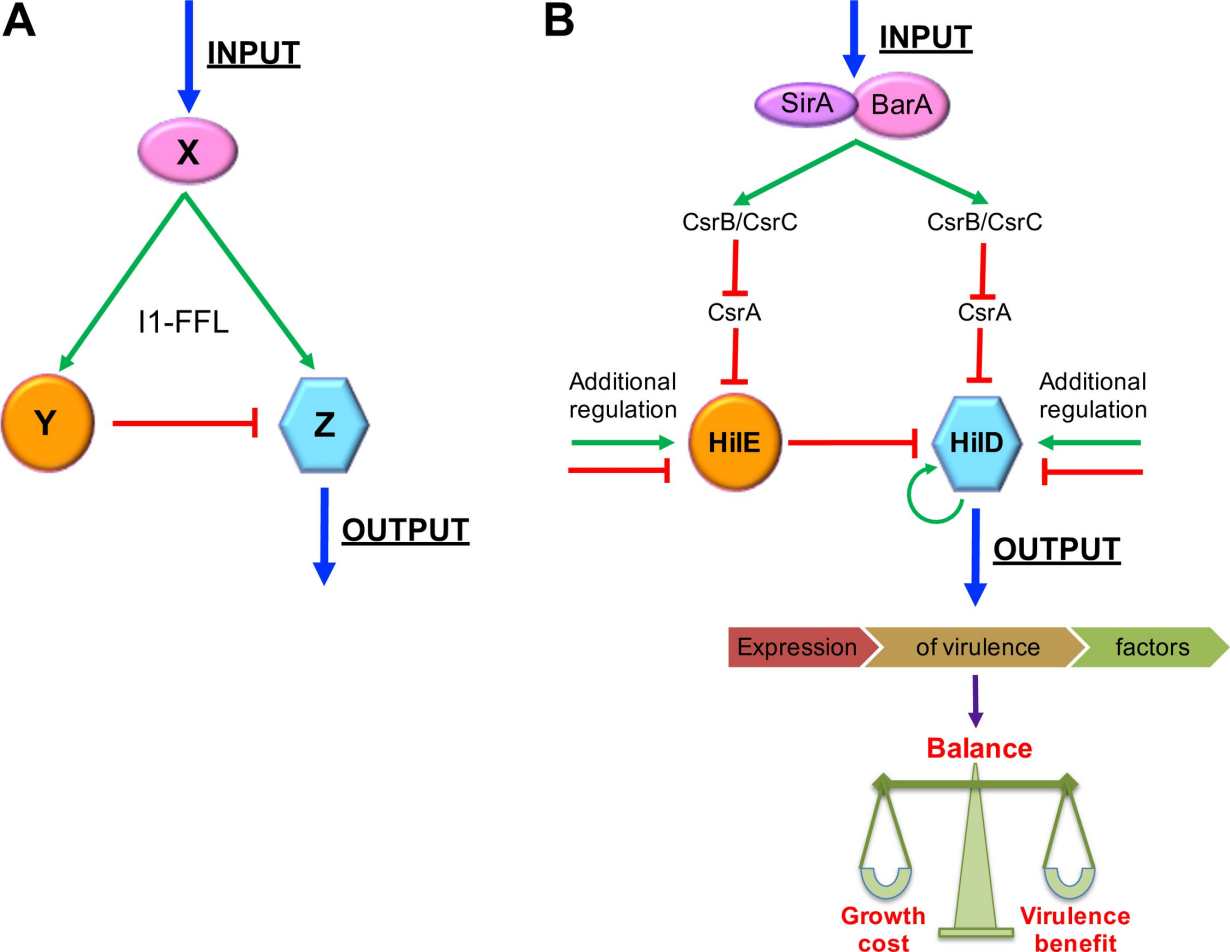
Structure and role of I1-FFL_SirA/BarA-HilE-HilD_ in *Salmonella* virulence. (A) Structure of the incoherent type-1 feedforward loop (I1-FFL) network motif, where the factor X activates factors Z and Y, and Y represses Z; thus, X exerts opposite controls on Z. (B) Structure and function of I1-FFL_SirA/BarA-HilE-HilD_. Molecules present in the mammalian intestines, such as short-chain fatty acids, have been shown to activate the SirA/BarA two-component system. SirA/BarA induces expression of both HilD and HilE, through the sRNAs CsrB and CsrC, which bind to CsrA and thus counteract CsrA-mediated repression on the *hilD* and *hilE* transcripts. HilE inhibits activity of HilD by protein-protein interaction. Therefore, SirA/BarA simultaneously exerts positive and negative control on HilD, which cooperates for the appropriate balance between the growth cost and the virulence benefit generated by the expression of tens of virulence genes regulated by HilD. Additional positive and negative regulation on HilD and HilE also would control the expression level of the genes regulated by HilD. See text for more details. Green arrows and red blunt-end lines indicate positive and negative control, respectively.

Regulatory networks are built up by recurring patterns of interactions between regulatory factors that are designed as network motifs, which are present in diverse organisms including bacteria, yeast, plants, animals, and humans [67,68]. One family of network motifs is the feedforward loop (FFL), where a factor X regulates factors Y and Z, and Y regulates Z. As the three regulatory interactions between X, Y, and Z can be either positive or negative, eight structural types of FFLs are possible [68,69]. One FFL commonly present in the best-studied transcriptional networks, those from *E*. *coli* and yeast, is the incoherent type-1 FFL (I1-FFL), where a factor X positively regulates factors Y and Z, while Y negatively regulates Z. Thus, X exerts an “incoherent” (or “paradoxical”) regulation on Z through two opposite effects [69,70] (Fig 8A), which represents a complex biological problem to decipher.

Our results from this study reveal a novel I1-FFL involved in gene expression in bacteria. We previously determined that in *S*. Typhimurium, the SirA/BarA two-component system induces the expression of the transcriptional regulator HilD, through the sRNAs CsrB and CsrC, which counteracts the CsrA-mediated repression of *hilD* translation [39]. HilD induces the expression of a large number of *Salmonella* virulence genes [2,15,26,29,31,32,66]. In this work, we show that the SirA/BarA/Csr system also regulates expression of HilE by a similar way. HilE negatively controls HilD activity by protein-protein interaction [36–38]. It is important to note that HilD is the only known target of HilE. Hence, the SirA/BarA-CsrB/C cascade exerts two opposite regulatory effects on HilD; the activation of the *hilD* mRNA translation, and the inhibition of HilD activity through HilE, thus constituting an I1-FFL (I1-FFL_SirA/BarA-HilE-HilD_) that involves regulation at the transcriptional, translational and post-translational levels (Fig 8B).

I1-FFLs can influence fold-change detection [71], adaptive tuning [72], response time [69,70] and response amplitude of gene expression [73]. Our results and those from a previous study [19] show that in the absence of HilE, the repressor factor of I1-FFL_SirA/BarA-HilE-HilD_ (Fig 8B), the growth of *S*. Typhimurium is slowed down in laboratory conditions, which can be attributed to the overexpression of genes regulated by HilD that has a deleterious effect by a still unknown mechanism. We found that the absence of HilE negatively affects the *S*. Typhimurium growth only in the presence of SirA and HilD, the two other components of I1-FFL_SirA/BarA-HilE-HilD_. Additionally, our results and a prior report [20] indicate that the SPI-1 genes are expressed *in vitro* in a bistable fashion that involves a control by HilE. In the presence of HilE only about one third fraction of the *S*. Typhimurium population expresses SPI-1, while in the absence of HilE the fraction of cells expressing SPI-1 increases more than twofold. Moreover, our results and those reported previously [20] indicate that the absence of HilE attenuates the intestinal colonization by *S*. Typhimurium, an effect also mediated through HilD. Interestingly, in the intestine of mice, the SPI-1 genes are also expressed in a bistable fashion, and in the absence of HilE, the proportion of *S*. Typhimurium cells expressing SPI-1 increases, imposing a growth pressure that leads to the selection of avirulent *hilD* mutants [20]. Furthermore, the lack of HilE increases the invasion of *S*. Typhimurium into epithelial culture cells by 2-5-fold [36,54]. Altogether, these findings suggest that I1-FFL_SirA/BarA-HilE-HilD_ is involved, together with other regulatory pathways, in the control of the precise amount and activity of HilD, resulting in an appropriate balance between the growth cost and the virulence benefit generated by the expression of the genes induced by this regulator (Fig 8B).

The SirA/BarA system seems to be directly activated by short-chain fatty acids such as acetate and formate, which are known to induce the expression of SPI-1 genes [8,74–76]. Therefore, these molecules commonly present in the mammalian intestines, probably in conjunction with other environmental cues, are expected to turn on I1-FFL_SirA/BarA-HilE-HilD_. However, several other positive and negative regulatory mechanisms acting on HilD or HilE provide additional inputs to I1-FFL_SirA/BarA-HilE-HilD_ for fine tuning the expression level of the genes regulated by HilD (Fig 8B). Expression of HilD is controlled positively by transcriptional autoregulation, directly or through the feedforward loop that HilD forms with HilC and RtsA [32–34,77,78]. HilD is also controlled by Hfq, ArcA, LoiA, Fis and Hu at the transcriptional initiation level [79–82], by Gre factors acting during transcriptional elongation [83], as well as by Fur and FliZ acting at the transcriptional/post-translational and post-translational levels, respectively [84,85]. Whereas the expression or amount of HilD is controlled negatively by H-NS, IscR, SsrB/SsrA and PhoP/PhoQ at the transcriptional level [79,86–89], by StdE/StdF at the post-transcriptional level [90], by FnrS and ArcZ at the translational level [13], by CRP-cAMP at the post-translational level [91], as well as by the Lon protease and CpxR/CpxA that decrease the stability of HilD [78,92]. Additionally, controversial reports indicate that the acetyltransferase enzyme Pat decreases or increases the stability and controls the DNA-binding activity of HilD [93–95]. On another hand, expression of HilE is controlled positively by FimYZ, PhoP/PhoQ, PhoB/PhoR and LeuO at the transcriptional level [56–59], whereas it is controlled negatively by MIc and IsrM at the transcriptional and translational levels, respectively [96]. Thus, the three main components of I1-FFL_SirA/BarA-HilE-HilD_ could connect in a dynamic way to this regulatory motif with the rest of the regulatory network controlling physiology and virulence in *Salmonella*.

Only a few I1-FFLs that control gene expression have been well-defined experimentally in bacteria. One of the best characterized is that involved in galactose metabolism in *E*. *coli*, where CRP-cAMP controls the expression of the *gal* and *mgl* genes by two opposite pathways, via direct positive regulation and negative regulation through GalS [70,73]. Additionally, in *Pseudomonas aeruginosa*, a quorum sensing system controlling virulence-related phenotypes is constituted by an I1-FFL, where LasR activates the expression of LasI, as well as that of RsaL, which in turn represses expression of LasI [97]. Furthermore, also in *P*. *aeruginosa*, the alternative sigma factor σ^22^ activates the expression of the virulence-associated enzyme AlgC, as well as that of the sRNA ErsA that represses translation of *algC* [98]. In addition, in *Rhodobacter sphaeroides*, two I1-FFLs are involved in the expression of photosynthesis-related genes. In one case, MppG exerts opposite transcriptional regulation on these genes, direct activation and indirect repression through AppA and PpsR, respectively [99], whereas in the other case, PrrA directly activates transcription of these genes but also represses their translation through the sRNA PcrZ [100,101]. Finally, in *S*. Typhimurium, the PhoP/PhoQ two-component system activates the expression of *mgtC*, encoding a virulence-associated protein, and that of the sRNA AmgR, while AmgR destabilizes *mgtC* mRNA [102], which also constitutes an I1-FFL.

Our study reveals a novel function for an I1-FFL, that is, fine tuning regulation to reduce the growth cost imposed by simultaneous expression of a high number of virulence genes. Our results and those from a previous study [20] provide evidence indicating that I1-FFL_SirA/BarA-HilE-HilD_ is necessary for intestinal colonization by *Salmonella*. Moreover, our findings illustrate the integration of ancestral (e.g. SirA/BarA) and acquired regulators (e.g. HilD and HilE) into a specific regulatory motif, which can lead to the expansion of regulatory networks during evolution.

## Materials and methods

### Ethics statement

Animal experiments were conducted according to the standard operating protocols approved by the International Committee for Animal Care and Use from CICUAL-UNAM and by the Official Mexican Norm NOM-062-Z00-1999.

### Bacterial strains, media and culture conditions

Bacterial strains used in this work are listed in Table 1. Bacterial cultures for β-galactosidase, chloramphenicol acetyltransferase and Western blot assays were grown in 250-ml flasks containing 50 ml of lysogeny broth (LB)-Miller, which were incubated at 37°C with shaking up to reach an O.D._600nm_ of 1.4 (5–8 h). Bacterial cultures for flow cytometry assays were grown in 16 x 150 mm glass tubes containing 5 ml of LB-Miller, which were incubated at 37°C with shaking up to reach an O.D._600nm_ of 1.2 (12–18 h). When necessary, culture medium was supplemented with ampicillin (200 μg/ml), streptomycin (100 μg/ml) or kanamycin (30 μg/ml).

**Table 1:** Bacterial strains and plasmids used in this study.

Strain or plasmid	Genotype or description	Source or reference
**Strain**		
*Salmonella* Typhimurium		
SL1344	Wild type; *xyl*, *hisG*, *rpsL*; Sm^R^	[114]
14028s	Wild type	ATCC
JPTM5	Δ*hilD*::*kan*	[15]
JPTM23	Δ*sirA*::*kan*	[39]
JPTM27	Δ*sirA*	[39]
JPTM39	Δ*barA*::*kan*	[39]
JPTM40	Δ*csrB*::*kan*	[39]
JPTM41	Δ*csrC*::*kan*	[39]
JPTM42	Δ*csrB*	[39]
JPTM43	Δ*csrB* Δ*csrC*::*kan*	[39]
DTM56	14028s Δ*hilE*::*kan*	[78]
DTM133	Δ*csrC*	This study
DTM134	Δ*csrB* Δ*csrC*	This study
DTM135	Δ*hilE*::*kan*	This study
DTM136	Δ*hilE*	This study
DTM137	Δ*hilE* Δ*hilD*::*kan*	This study
DTM138	14028s *hilE*::*3XFLAG-kan*	This study
DTM139	14028s *hilE*::*3XFLAG*	This study
CJ035	*sirA*::Tn*10*d	[40]
DTM140	*sirA*::Tn*10*d	This study
DTM141	Δ*hilE sirA*::Tn*10*d	This study
*Escherichia coli*		
DH10β	Laboratory strain	(Invitrogen)
**Plasmids**		
pKK232-8	pBR322 ori vector containing a promoterless chloramphenicol acetyltransferase (*cat*) gene, Ap^R^	[115]
philA-cat	pKK232-8 derivative containing a *hilA-cat* transcriptional fusion from nucleotides -410 to +446	[15]
pinvF-cat	pKK232-8 derivative containing an *invF-cat* transcriptional fusion from nucleotides -306 to +213	[15]
ppipB-cat	pKK232-8 derivative containing a *pipB-cat* transcriptional fusion from nucleotides -737 to +70	[15]
psopB-cat	pKK232-8 derivative containing a *sopB-cat* transcriptional fusion from nucleotides -400 to +128	[15]
pssaG-cat	pKK232-8 derivative containing a *ssaG-cat* transcriptional fusion from nucleotides -232 to +361	[15]
pssrAB-cat	pKK232-8 derivative containing a *ssrAB-cat* transcriptional fusion from nucleotides -303 to +3054	[15]
pMPMA3ΔPlac null-*gfp*[LVA]/R	P15A ori vector containing a promoterless *gfp* reporter gene encoding a destabilized variant of green fluorescent protein, GFP [LVA], Ap^R^	[116]
pMPMA3ΔPlac P*invF*-*gfp*[LVA]/R	pMPMA3ΔPlac null-*gfp*[LVA]/R derivative containing an *invF-gfp* transcriptional fusion	[116]
pRS414	pBR322 ori vector for the construction of translational fusions to the *lacZ* reporter gene, Ap^R^	[103]
philE-lacZ	pRS414 derivative containing a *hilE-lacZ* translational fusion from nucleotides -424 to +49	This study
pMPM-K3	P15A ori cloning vector, *lac* promoter, Kan^R^	[104]
pK3-CsrA	pMPM-K3 derivative expressing CsrA from the *lac* promoter	[39]
pK3-CsrB	pMPM-K3 derivative expressing CsrB from the *lac* promoter	[39]
pK3-SirA	pMPM-K3 derivative expressing SirA from the *lac* promoter	[39]
pMPM-K4Ω	ColE1 ori vector, Kan^R^	[104]
pK4-SirA	pMPM-K4Ω derivative expressing *sirA* from its own promoter	This study
pMPM-K6Ω	p15A ori vector, *ara* promoter, Kan^R^	[104]
pK6-HilD	pMPM-K6Ω derivative expressing HilD from the *ara* promoter, Kan^R^	[31]
pK6-HilE	pMPM-K6Ω derivative expressing HilE from the *ara* promoter, Kan^R^	[38]
pCP20	Plasmid expressing FLP recombinase from a temperature-inducible promoter, Ap^R^	[106]
pSUB11	pGP704 derivative template plasmid for FLAG epitope tagging	[105]

The coordinates of the nucleotides for the *cat* and *lacZ* fusions are relative to the transcriptional start site reported for each gene. Ap^R^, ampicillin resistance; Kan^R^, kanamycin resistance; Sm^R^, streptomycin resistance.

### Plasmids

Table 1 shows the plasmids used in this study. To construct the philE-lacZ plasmid, a DNA fragment spanning the full-length intergenic region upstream *hilE* and the first ten codons of this gene, was amplified by PCR with the primers HilE-EcoRI-Fw1 (5’-TTAAATGAATTCACATAGTAAATATGTTCTATTG-3’) and HilE-BamHI-Rev1 (5’-GTTTTCAAGGATCCTGATCCGGCTTTCGCCTTC-3’), containing sites for EcoRI and BamHI restriction enzymes, respectively (underlined in the sequences). The PCR product was digested with EcoRI and BamHI and ligated into the pRS414 vector [103] digested with the same enzymes. To construct the pK4-SirA plasmid, a DNA fragment spanning *sirA* and its upstream intergenic region was amplified by PCR with the primers SirAF (5’-GCCGGATCCATCGCCTGCAGCATCAGC-3’) and SirARgfp (5’-ACCAAGCTTGTCATACATACGATAGACACCG-3’), containing sites for BamHI and HindIII restriction enzymes, respectively (underlined in the sequences). The PCR product was digested with BamHI and HindIII and ligated into the pMPM-K4Ω vector [104] digested with the same enzymes. The constructed recombinant plasmids were characterized by PCR amplification and sequencing.

### Construction of mutants and strains expressing FLAG-tagged proteins

P22 transduction was used to transfer the Δ*hilE*::*kan* allele from DTM56 into the WT *S*. Typhimurium SL1344 strain, generating DTM135, to transfer the Δ*hilD*::*kan* allele from JPTM5 into DTM136, generating DTM137, and to transfer the *sirA*::Tn*10*d allele from CJ035 into the WT *S*. Typhimurium SL1344 and DTM136 strains, generating DTM140 and DTM141, respectively. The chromosomal *hilE* gene was FLAG-tagged in the WT *S*. Typhimurium 14028s strain by a previously reported method based on the λRed recombinase system [105], using the primers HilE-FLAG-5’ (5’-AGCAAAACACGGCGGCCTCTTCACCGACAGGCGCTGTGGCGAGACTACAAAGACCATGACGG-3’) and HilE-FLAG-3’ (5’-ATACAGCATCGCCCACTGCGAGTCCGCAAGCTTGTTTTGTCCCATATGAATATCCTCCTTAG-3’), generating DTM138; the sequences corresponding to the pSUB11 template plasmid are underlined. The kanamycin resistance cassette was excised from the JPTM41, JPTM43, DTM135 and DTM138 strains, by using helper plasmid pCP20 expressing the FLP recombinase, as described previously [106], generating strains DTM133, DTM134, DTM136 and DTM139, respectively. All modified strains were verified by PCR amplification and sequencing.

### Enzymatic assays

β-galactosidase and chloramphenicol acetyltransferase (CAT) assays, as well as protein quantification to calculate the respective enzymatic specific activity, were performed as described previously [107,108].

### Flow cytometry analysis

Samples containing ~10^7^ cells were taken from bacterial cultures grown as described above. After washing with 0.22-μm-pore-size filtered 1X phosphate-buffered saline (PBS), cells were fixed for 20 min at room temperature in 400 μl of 2% (wt/vol) paraformaldehyde (Sigma) in 1X PBS. Fixed cells were centrifuged and resuspended in one ml of 1X PBS. GFP fluorescence was assessed on a FACSCanto II cytometer (BD Biosciences) equipped with the FACSDiva software (BD Biosciences). Data from 100 000 events were analyzed with the FlowJo v10 software (Tree Star Inc). Cells carrying the *gfp* reporter gene without a promoter were used to define the background level of fluorescence. Data shown represent the frequency of GFP positive cells.

### Western blotting

Preparation and visualization of immunoblots were performed as described previously [88]. Monoclonal anti-FLAG M2 (Sigma) or polyclonal anti-GroEL (StressGen) antibodies were used at 1:3,000 and 1:100,000 dilutions, respectively. Horseradish peroxidase-conjugated anti-mouse or anti-rabbit antibodies (Pierce) diluted at 1:10,000 were used as secondary antibodies.

### Electrophoretic mobility shift assays

The electrophoretic mobility shift assays (EMSAs) followed published procedures [109,110]. His-tagged CsrA (CsrA-H6) from *E*. *coli* was purified as described previously [111]. Note that CsrA from *E*. *coli* and *S*. Typhimurium are identical. RNA was synthesized *in vitro* using the RNA Maxx Transcription Kit (Agilent Technologies). PCR fragments used as templates in transcription reactions contained a T7 promoter and *hilE* sequence extending from +153 to +280 relative to the start of transcription. Gel-purified RNA was 5′-end labeled with [γ-^32^P]-ATP (7,000 Ci/mmol). RNA suspended in Tris-EDTA (TE) buffer was heated to 85°C for 3 min followed by slow cooling at room temperature for 10 min. Binding reactions (10 μl) contained 10 mM Tris-HCl, pH 7.5, 10 mM MgCl_2_, 100 mM KCl, 200 ng/μl yeast RNA, 0.2 mg/ml bovine serum albumin (BSA), 7.5% glycerol, 20 mM dithiothreitol (DTT), 0.2 nM RNA, CsrA-H6 (various concentrations), and 0.1 mg/ml xylene cyanol. Competition assay mixtures also contained unlabeled competitor RNA. Reaction mixtures were incubated for 30 min at 37°C to allow CsrA-RNA complex formation. Samples were then fractionated through native 10% polyacrylamide gels using 0.5X Tris-borate-EDTA (TBE) as the gel running buffer. Radioactive bands were visualized with a Typhoon 9410 phosphorimager (GE Healthcare) and quantified using ImageQuant 5.2 software. Apparent equilibrium binding constants (*K*_*d*_) of CsrA-*hilE* RNA interaction were calculated as described previously [112].

### Competitive growth experiment

The WT *S*. Typhimurium SL1344 strain and its isogenic mutants were grown in 5 ml of LB at 37°C with shaking, until the cultures reached an OD_600nm_ of 0.6. Next, mixed cultures were started by inoculating an equal amount of the initial culture of the respective strains (500 μl + 500 μl) in 250-ml flasks containing 50 ml of fresh LB without antibiotics, which were incubated at 37°C with shaking for 8 h. At 0, 2, 4, 6 and 8 h, culture samples were taken to determine colony forming units (CFUs) of each strain, by plating serial dilutions in 1X PBS on LB agar supplemented with the indicated antibiotics. For the WT+Δ*hilE*::*kan*, WT+Δ*hilD*::*kan* and WT+Δ*hilE* Δ*hilD*::*kan* mixed cultures, CFUs of the Δ*hilE*::*kan*, Δ*hilD*::*kan* or Δ*hilE* Δ*hilD*::*kan* mutants (kanamycin and streptomycin resistant) were obtained directly from the count on LB agar + kanamycin/streptomycin plates, whereas CFUs of the WT strain (streptomycin resistant, kanamycin susceptible) were determined by subtracting the number of CFUs on LB agar + kanamycin/streptomycin plates from that of CFUs on LB agar + streptomycin plates. For the Δ*sirA*/pMPM-K3+Δ*hilE sirA*::Tn*10*d/pMPM-K3 and Δ*sirA*/pK3-SirA+Δ*hilE sirA*::Tn*10*d/pK3-SirA mixed cultures, CFUs of the Δ*hilE sirA*::Tn*10*d/pMPM-K3 and Δ*hilE sirA*::Tn*10*d/pK3-SirA strains (kanamycin, tetracycline and streptomycin resistant) were obtained directly from the count on LB agar + kanamycin/tetracycline/streptomycin plates, whereas CFUs of the Δ*sirA*/pMPM-K3 and Δ*sirA*/pK3-SirA strains (kanamycin and streptomycin resistant, tetracycline susceptible) were determined by subtracting the number of CFUs on LB agar + kanamycin/tetracycline/streptomycin plates from that of CFUs on LB agar + kanamycin/streptomycin plates. To compare the growth of the two strains in the respective mixed culture (competitive growth), the number of CFUs for each strain is indicated as a fraction (percentage).

### Competitive index assay

Pathogen-free BALB/c female mice (6- to 7-week-old) were obtained from the Experimental Medicine Research Unit, School of Medicine, UNAM, México. Maintenance, streptomycin treatment (50 mg) and euthanasia of the mice were performed as described previously [113]. Overnight cultures of the WT *S*. Typhimurium SL1344 strain and its isogenic Δ*hilE*, Δ*hilD* and Δ*hilE* Δ*hilD* mutants were diluted 1:100 in 5 ml of fresh LB and incubated at 37°C with shaking for about 3 h. Then, bacterial suspensions containing 0.5 x 10^7^ CFUs/ml of each the WT strain and the respective mutant (WT + Δ*hilE*, WT + Δ*hilD* or WT + Δ*hilE* Δ*hilD*) were prepared in 1X PBS. Next, groups of eight streptomycin-pretreated mice were infected by orogastric route with 100 μl of the corresponding bacterial suspension. At 3 days post-infection, cecum and feces were harvested aseptically and homogenized in 1 ml of sterile and cold 1X PBS, solubilized in 2% Triton X-100, serially diluted and differentially plated on LB agar plates containing streptomycin or kanamycin to determine CFUs/mg. The number of CFUs for each strain was obtained as described in Competitive growth experiment. The competitive index was calculated by dividing the total CFUs of mutant strain by the total CFUs of the WT strain.

### Statistical analysis

Data analyses were performed using GraphPad Prism version 6.0c for Mac OS X, GraphPad Software, La Jolla, California USA, using One-way ANOVA followed by Tukey’s multiple comparisons posttest. *P*-values < 0.05 were considered statistically significant.
